# Chemoattractive potential of stem cells from apical papilla in peripheral blood monocytes: an in vitro study

**DOI:** 10.1590/0103-644020256694

**Published:** 2026-01-19

**Authors:** Alexandre Guimarães dos Santos, Letícia Martins Santos, Juliana Garuba Rahhal, Mariane Sloniak, Cristina Cunha Villar, Fernando Neves Nogueira, Carla Renata Sipert

**Affiliations:** 1 Departamento de Dentística, Faculdade de Odontologia, Universidade de São Paulo, São Paulo Brasil; 2 Divisão de Periodontia, Departamento de Estomatologia, Faculdade de Odontologia, Universidade de São Paulo, São Paulo Brasil; 3 Departamento de Biomateriais e Biologia Oral, Faculdade de Odontologia, Universidade de São Paulo, São Paulo Brasil

**Keywords:** stem cells, chemokines, monocytes

## Abstract

Chemokine (CC-motif) ligand 2 (CCL2) is the primary chemokine involved in monocyte migration from peripheral blood to tissues. These cells, in turn, will differentiate into macrophages and osteoclasts. Given that apical papilla stem cells (SCAP) are an important source of CCL2, this study aimed to investigate whether SCAP culture supernatant can recruit monocytes in vitro via CCL2. SCAP-conditioned medium (CM-SCAP) was obtained through primary culture after confirming the osteo/odontogenic differentiation potential of these cells. The supernatant was analyzed for CCL2 using an immunoenzymatic assay. Monocytes were isolated from human peripheral blood and positively selected using magnetic beads. CD 14+ cells were seeded into 5-μm pore transwell inserts placed in wells containing CM-SCAP. After 24 hours, the insert was removed, and the migrated cells were quantified using the Alamar Blue viability assay. Recombinant human CCL2 was used as a positive control, while the proliferation medium only was used as the negative control. CM-SCAP in the presence of a CCL2 neutralizer antibody was also tested. Statistical analysis was performed to assess group differences, considering data normality and a 5% significance level (p < 0.05). SCAP produced CCL2 in vitro, and none of the CM-SCAP dilutions affected monocyte viability. Wells containing CM-SCAP displayed a significant increase in cell migration compared to the control. This finding was abrogated by the CCL2 neutralizing antibody. Therefore, SCAP supernatant can induce monocyte migration in vitro through a CCL2-dependent mechanism.

## Resumo

CCL2 (*“Chemokine (CC-motif) ligand 2”*) é a principal quimiocina envolvida na migração de monócitos do sangue periférico para o tecido. Tais células, por sua vez, irão se diferenciar em macrófagos e osteoclastos. Considerando que as células-tronco da papila apical (SCAP) são uma importante fonte de CCL2, este estudo teve como objetivo investigar se o sobrenadante de cultura de SCAP é capaz de recrutar monócitos in vitro, através de CCL2. O meio condicionado por SCAP (MC-SCAP) foi obtido através de cultura primária após confirmado o potencial de diferenciação osteo/odontogênico destas células. O sobrenadante foi sujeito a quantificação de CCL2 usando ensaio imunoenzimático. Monócitos foram obtidos por meio de sangue humano periférico e isolados por seleção positiva por *“bead”* magnética. Células CD 14+ foram plaqueadas em insetos *“transwells”* com poros de 5-μm que foram posicionados sobre poços contendo MC-SCAP. Após 24 horas, o inserto foi removido e as células que migraram foram quantificadas por ensaio de viabilidade Alamar Blue. CCL2 recombinante humana foi utilizada como controle positivo, enquanto que o meio de proliferação somente foi utilizado como controle negativo. MC-SCAP na presença de um anticorpo neutralizante de CCL2 também foi testado. A análise estatística foi realizada considerando a normalidade dos dados, com nível de significância a 5% (p < 0.05) para avaliar diferenças entre grupos. SCAP foram capazes de produzir CCL2 *in vitro* e nenhuma das concentrações de MC-SCAP afetaram a viabilidade de monócitos. Poços contendo MC-SCAP mostraram um aumento significativo de migração celular comparado ao controle. Esses achados foram revertidos pelo anticorpo neutralizante de CCL2. Dessa maneira, o sobrenadante de SCAP pode induzir a migração de monócitos *in vitro*, através de um mecanismo CCL2 dependente.

**Figure:**
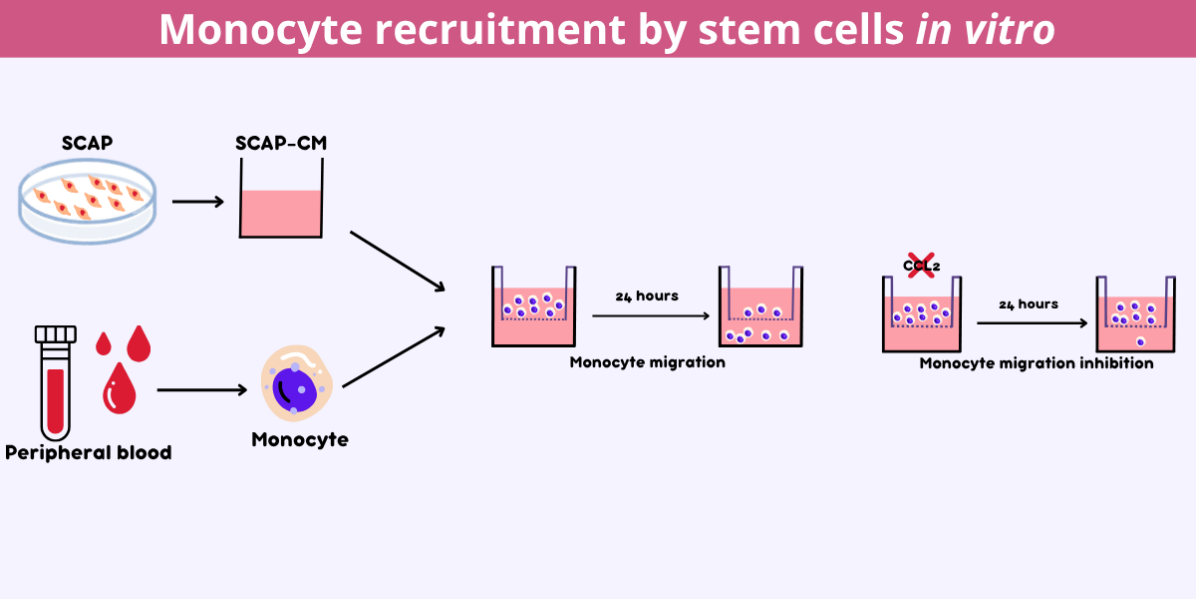

## Introduction

The Regenerative Endodontic Treatment (RET), introduced by Banchs and Trope in 2004 ^1^, is the current gold standard for root canal therapy in immature permanent teeth with pulp necrosis ^2^. With the increasing success of RET, this approach has also been proposed for mature teeth with apical periodontitis ^3^. Although studies remain limited, RET appears to be a viable alternative for these cases as well ^4^. To improve RET and other regenerative procedures, some recent studies developed scaffolds loaded with stem cells or not aiming to guide the bone, cartilage, or pulp regeneration process ^5^ ^), (^ ^6^ ^), (^ ^7^. Even though this approach is promising, the lack of knowledge about stem cells' behaviour makes it difficult to understand and interfere in the tissue neoformation.

In RET, this process is primarily due to the activity of stem cells from the apical papilla (SCAP) ^8^ ^,^ ^9^ ^).^ These cells exhibit significant differentiation and proliferation potential ^6^, playing a role in modulating the immune response by either activating or suppressing specific immune cells ^10^ ^), (^ ^11^ ^), (^ ^12^ ^), (^ ^13^. This type of interaction may affect the proliferation and differentiation of surrounding cells, facilitating the regeneration process ^7^.

In cases of pulp necrosis, the inflammatory response attracts monocytes, which differentiate into phagocytic cells such as macrophages, osteoclasts, and odontoclasts ^14^. Although this process is essential for host protection, if dysregulated, it may lead to bone or tooth resorption ^13^. The migration of these cells is directed by chemokines, which are cytokines with chemotactic properties. CCL2 (Chemokine (CC-motif) ligand 2), also known as Monocyte Chemoattractant Protein-1 (MCP-1), was the first chemokine to be purified and remains among the most extensively studied due to its central role in monocyte recruitment and regulation in immune responses across various diseases ^5^ ^), (^ ^16^ ^), (^ ^17^.

Given that SCAP is a known source of cytokines, including CCL2 ^10^ ^), (^ ^18^ ^), (^ ^19^ ^), (^ ^20^, this study aims to assess whether SCAP can induce chemotaxis of peripheral human blood monocytes in vitro.

## Materials and methods

### Human stem cells from apical papilla culture

This study was approved by the local Ethics Committee (Process 20086119.4.0000.0075) and conducted in accordance with the Declaration of Helsinki. SCAPs were obtained from an immature third molar by explant technique, as previously described ^21^, after the participant signed an informed consent form following tooth extraction. 

The human apical papilla was removed from the root, and a primary cell culture was established. Cells were maintained in a proliferation medium composed of α-Modified Eagle Medium (α-MEM) (Gibco), supplemented with 10% fetal bovine serum (FBS) (Gibco), and 2mM L-glutamine (Invitrogen, Life Technologies, Carlsbad, EUA), 100µg L-ascorbate-2-phosphate (Sigma-Aldrich, St. Louis, EUA), 100 U/mL penicillin, and 100 mg/ mL streptomycin (GIBCO/Invitrogen) at 37^o^C in a 5% CO_2_ atmosphere.

### Human stem cells from apical papilla characterization

SCAP were previously characterized morphologically and functionally by our research group ^20^. To prove the osteo/odontogenic differentiation potential, Alizarin Red Staining was performed using a differentiation medium that was replaced every two days and was composed of the addition of 10 nmol/L of dexamethasone (Invitrogen), 10 mmol/L of β-glycerophosphate (Sigma-Aldrich), and 50 μg/mL of ascorbic acid (Sigma-Aldrich) in the proliferation media. The mineralization deposit was investigated after 28 days using 40 mmol/L alizarin red S staining (CatA5533, Sigma-Aldrich) at a pH of 4.2 to stain the SCAPS, and 500 µl of Ammonium Hydroxide (NH_4_OH) was added for quantification analysis in a spectrophotometer (Biotek Synergy HT) at 405 nm.

### Conditioned medium preparation

The Conditioned Medium from SCAP (CM-SCAP) was collected from cells at the 5th passage. Cells were detached with trypsin EDTA (0.25%) (Invitrogen), seeded in a 24-well plate at a concentration of 5 × 10_4_ cells per well with α-MEM 10%FBS, and incubated at 37^o^C in 5% CO_2_. The medium was replaced every 24 hours for 72 hours with FBS-free α-MEM. Afterwards, the supernatant was collected and centrifuged at 800 x g/ 10 minutes / 4 °C, and stored at -80 °C.

### CCL2 quantification

CCL2 concentration in CM-SCAP was analyzed by Enzyme-linked Immunosorbent Assay (ELISA), using DuoSet kits (R&D Systems - DY279-05) following the manufacturer's instructions. The reading was performed in a spectrophotometer (Biotek Synergy HT) at 450 nm. CCL2 concentration was normalized by quantification of total proteins by Lowry’s Method ^22^. 

### Isolation of Human Peripheral Blood Mononuclear Cells

Human Peripheral Blood Mononuclear Cells (PBMC) were obtained from the peripheral blood of three healthy donors who previously signed an informed consent form. Blood was diluted in PBS 1:1 and added to Ficoll-Plaque TM Premium (GE Healthcare). Then the solution was centrifuged at 400 x g/ 30 minutes without a break, and PBMC was collected. 

### Isolation of CD14+ cells

Cells CD14+ were separated from PBMC cells using positive selection with micromagnetic beads from MACS monocyte isolation kits, following the manufacturer’s instructions (Miltenyi Biotec). 

### CM-SCAP cytotoxicity assay

The cytotoxic potential from CM-SCAP in monocytes was tested using the MTT (3-[4,5-dimethylthiazol-2-yl]-2,5-diphenyltetrazolium bromide) assay. CD14+ cells were plated at 1.25 X 10_4_ cells/well in a 96-well plate and incubated in α-MEM with 10% FBS for 24 hours. After that, the medium was removed, and cells were stimulated with decreasing dilutions of CM-SCAP (½, ¼, and 1/8) for 24 hours. The MTT solution (1:10 in PBS) was added to each well, and the plate was incubated for 4 hours at 37°C in a 5% CO_2_ atmosphere. Then the solution was removed, 200 µL of dimethyl sulfoxide (DMSO) was added to each well. The absorbance was measured in a spectrophotometer (Synergy H1, Biotek) at a wavelength of 570 nm.

### Migration assay

CD14+ cells were plated in the upper chamber of a 24-transwell plate across 5-μm polycarbonate membranes (Corning Incorporated, USA) at a density of 5 × 10_4_ cells/transwell in the presence of α-MEM 10%. CM-SCAP diluted ½ in α-MEM with 10% FBS was added to the lower chamber. To evaluate the influence of CCL2 on chemotaxis, wells containing CM-SCAP in the presence of neutralizing human CCL2 antibody (MAB679-SP, R&D Systems) were also tested. 300 ng/ml of Recombinant Human CCL2 (279-MC-010 R&D Systems) was used for positive control, while α-MEM 10% was the negative control. After 24 hours, the transwell insert was discarded, and Alamar blue solution was added in the proportion of 1:10 in the lower chambers to quantify monocyte migration. The plate was incubated for 4 hours, protected from the light, and the fluorescence was measured in a spectrophotometer (Synergy H1, Biotek) at an emission wavelength of 590 nm and excitation at 530 nm. 

### Statistical analysis

Data was analyzed according to the normal distribution evaluated by the Shapiro-Wilk test. A one-way analysis of variance (ANOVA), followed by Tukey's post-test, was used for statistical analysis. Significance was set at p < 0.05, and the analysis was performed using GraphPad Prism 9.0 (GraphPad Software, San Diego, CA, USA).

## Results

### Human stem cells from apical papilla characterization

**Figure 1:**
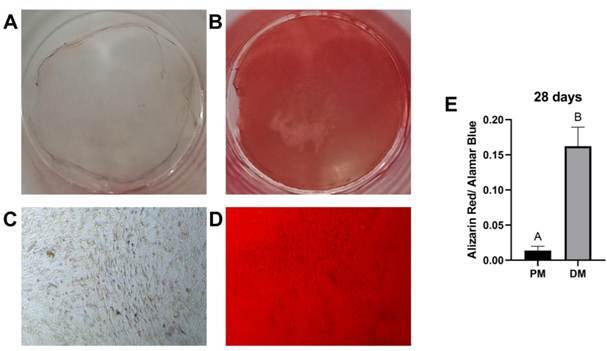
Osteo/odontogenic mineralization potential. An alizarin red S staining assay was performed to analyze the calcium deposition of SCAP for functional characterization. Cells were maintained in proliferation (A and C) or differentiation (B and D) medium at the 28-day time point. Macroscopic view (A and B). Microscopic view (C and D). Semi-analytical densitometry of calcium standardized by the Alamar Blue (E) produced by SCAP stimulated with proliferation medium (PM) or differentiation medium (DM). The results showed the mean and standard deviation of the experiment performed in triplicate. Different letters indicate statistical differences between groups (simple T-test, unpaired, p < 0.05).

After 28 days, SCAP stimulated with differentiation medium presented significantly increased mineralization compared to proliferation medium according to Alizarin Red S staining assay (Figure 1A-D). Semi-analytical densitometry revealed a statistically significant increase in osteo/odontogenic differentiation potential in differentiation medium wells (Figure 1E). Previous studies confirmed that cells were positive for CD146 and STRO-1 and negative for CD45 and CD34 in flow cytometry ^20^. 

### CCL2 quantification and CM-SCAP cytotoxicity assay

**Figure 2:**
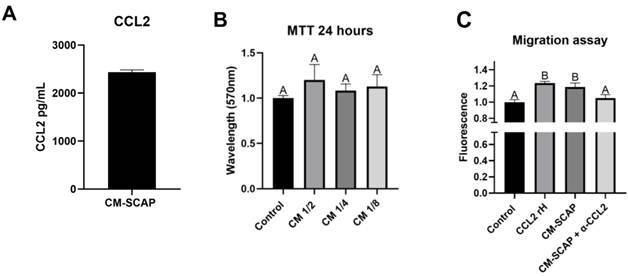
Production of CCL2 quantified by ELISA assay in SCAP conditioned medium (CM-SCAP). Bars show the mean and standard deviation of the experiment (N=3) (A). Viability of monocytes stimulated with decreasing dilutions of SCAP supernatant (CM) compared with control (proliferation medium alone). Absorbance (570nm) data obtained from the MTT assay after 24 h of stimulus exposure (B). Fluorescence data obtained by Alamar Blue assay at experimental time of 24 h. Conditioned medium by SCAP (CM-SCAP) diluted ½ in the presence or not of a CCL2 neutralizer (CM-SCAP + α-CCL2) compared to control (proliferation medium alone) and Recombinant Human CCL2 (CCL2 rH) (C). The result showed the mean and standard deviation of the experiments performed in triplicate. Different letters represent statistical differences between groups. (One-Way ANOVA with Tukey's test, p<0.05) (B and C).

2439,1933 pg/ml of CCL2 was quantified in CM-SCAP (Figure 2A). None of the CM-SCAP dilutions tested altered the viability of CD 14+ cells after 24 hours of exposure (Figure 2B). Based on these findings, a ½ dilution of CM-SCAP was chosen for the following experiments.

### Migration assay

Wells containing CM-SCAP presented similar migration patterns compared to the positive control (Recombinant Human CCL2). This effect was abrogated in the presence of a CCL2 neutralizer antibody, leading to levels close to negative control (Figure 2C). This assay was repeated 3 times in triplicate.

## Discussion

Stem cells from the apical papilla (SCAP) play a crucial role in root formation ^23^; however, in cases of pulp necrosis or pulpitis, this process can be disrupted ^24^. Regenerative Endodontic Treatment (RET) is currently the most effective treatment for necrosis in immature teeth, although the mechanisms of tissue neoformation remain incompletely understood. Consequently, SCAP’s potential to modulate the immune response has become an area of interest in Endodontics. 

 The capacity of tissue regeneration of resident cells depends on the number of defense cells, cytokine production, intensity, and duration of injury, with a high concentration of leukocytes able to inhibit the tissue repair process, resulting in necrosis ^5^. Recent studies showed the ability of bacteria to invade SCAP and disturb the mineralization and differentiation of these cells ^9^; however, little is known about SCAP's potential for self-defense, mainly about the ability to recruit immune cells.

Even though studies on the impact of SCAP on other cell populations have shown mixed outcomes, the production of CCL2 by SCAP has been confirmed, particularly with increased production in response to bacterial metabolites ^19^ ^); (^ ^20^.

Monocytes are essential leukocytes in tissue repair and host defense, as they differentiate into various phagocytes such as macrophages and osteoclasts, particularly during inflammation ^25^ ^,^ ^26^. This differentiation supports the immune response to infectious agents and contributes to tissue integrity. When tissue injury occurs, monocytes are recruited to the affected area by inflammatory chemokines, which initiate a defensive response but may also lead to bone or tooth resorption if dysregulated ^27^. The migration of monocytes is vital for maintaining bone metabolism, which requires a balance between osteoblast and osteoclast activity; osteoclasts, in particular, result from monocyte differentiation ^19^ ^),(^ ^28^ ^),(^ ^29^. Consequently, the presence of an increased number of monocytes in the periapical tooth area, occasioned by pulp necrosis, will not necessarily result in bone resorption. The mechanisms of monocyte differentiation in osteoclasts require the presence of metabolites in the inflamed tissue that will further induce increased differentiation of osteoclasts, such as macrophage colony-stimulating factor (M-CSF) and receptor activator of nuclear factor kappa-Β ligand (RANKL) ^27^.

The monocyte migration is primarily driven by CCL2 ^15^, although chemokine-cell interactions are not exclusive. For instance, CCL2 also attracts NK cells and specific T lymphocytes ^15^, while monocytes may be attracted by other chemokines, including CCL4, CCL5, CCL7, and CCL8 ^15^ ^),(^ ^30^.

At the migration assay of the present study, the positive control presented over 200 times more CCL2 than CM-SCAP, yet both induced statistically similar monocyte migration. This suggests that additional metabolites from SCAP may be contributing to migration. However, CM-SCAP with a CCL2 neutralizer antibody failed to induce CD14+ cell migration, indicating that CCL2 produced by SCAP is essential for monocyte recruitment. We hypothesize that these SCAP metabolites work synergistically with CCL2, producing a lower migration effect in its absence, consistent with previously observed chemokine synergies ^31^. Although limited, studies indicate that SCAP can produce other chemokines capable of inducing monocyte migration. For example, Takimoto et al. ^32^ found that SCAP exposed to E. coli lipopolysaccharides increased CCL5 gene expression. SCAP also secretes interleukin 8 (IL-8, or CXCL8), which recruits neutrophils ^33^ ^,^ ^34^, suggesting that SCAP may recruit diverse leukocyte types as needed. 

Although being an in vitro study, the results shown in this article must be analyzed carefully, and some limitations are present. In this methodology, we assessed the production of CCL2 by SCAP in a physiologic condition, but the presence of bacterial metabolites in the root canal could improve the production of cytokines, as previously demonstrated ^10^ ^), (^ ^19^, changing the intensity of the monocyte migration in a clinical scenario. In addition, besides SCAP showing potential to attract monocytes, this study did not investigate the influence of the apical papilla in the differentiation of these cells. Further studies should analyze whether the presence of SCAP will interfere with macrophage or osteo/odontoclast differentiation, leading to a higher immune response or tissue resorption.

The use of SCAP in regenerative procedures is an increasing area of interest in the medical field, with some studies developing scaffolds loaded with SCAP capable of promoting cartilage repair in vivo, with the aim of further solving clinical problems such as mandibular retraction ^35^. Moreover, studies with this approach are extensively documented in scientific literature. Scaffolds with dental stem cells are being studied to improve bone, cartilage, pulp, and dentinal repair in vitro, with promising results, although clinical studies are still scarce ^5^. The results obtained in this study help us to understand the capacity of SCAP to modulate the microenvironment recruiting leukocytes in physiological conditions, and help to improve the knowledge regarding clinical approaches using these cells. 

Therefore, this study suggests that SCAP recruits monocytes during root formation, even under physiological conditions, without showing cytotoxic potential. These findings enhance our understanding of SCAP's role in immune modulation, contributing to insights into RET mechanisms and the effects of pulp pathologies in immature teeth.

## Conclusion

In conclusion, the results of this study showed that SCAP can induce migration of monocytes in vitro by a CCL2-dependent mechanism.

## Data Availability

The research data are available upon request.
